# Implementation of a Cooking Bus intervention to support cooking in schools in Wales, UK

**DOI:** 10.1108/he-06-2014-0073

**Published:** 2017

**Authors:** Jeremy Segrott, Jo Holliday, Simon Murphy, Sarah Macdonald, Joan Roberts, Laurence Moore, Ceri Phillips

**Affiliations:** Centre for Trials Research, Centre for the Development and Evaluation of Complex Interventions for Public Health Improvement (DECIPHer), Cardiff University, Cardiff, UK; Centre for the Development and Evaluation of Complex Interventions for Public Health Improvement (DECIPHer), Cardiff School of Social Sciences, Cardiff University, Cardiff, UK; MRC/CSO Social and Public Health Sciences Unit, University of Glasgow, Glasgow, UK; Swansea Centre for Health Economics, College of Human and Health Sciences, Swansea University, Swansea, UK

**Keywords:** Teachers, Food, Children, School health promotion, Behavioural change, Inter-agency liaison

## Abstract

**Purpose:**

The teaching of cooking is an important aspect of school-based efforts to promote healthy diets among children, and is frequently done by external agencies. Within a limited evidence base relating to cooking interventions in schools, there are important questions about how interventions are integrated within school settings. The purpose of this paper is to examine how a mobile classroom (Cooking Bus) sought to strengthen connections between schools and cooking, and drawing on the concept of the sociotechnical network, theorise the interactions between the Bus and school contexts.

**Design/methodology/approach:**

Methods comprised a postal questionnaire to 76 schools which had received a Bus visit, and case studies of the Bus’ work in five schools, including a range of school sizes and urban/rural locations. Case studies comprised observation of Cooking Bus sessions, and interviews with school staff.

**Findings:**

The Cooking Bus forged connections with schools through aligning intervention and schools’ goals, focussing on pupils’ cooking skills, training teachers and contributing to schools’ existing cooking-related activities. The Bus expanded its sociotechnical network through post-visit integration of cooking activities within schools, particularly teachers’ use of intervention cooking kits.

**Research limitations/implications:**

The paper highlights the need for research on the long-term impacts of school cooking interventions, and better understanding of the interaction between interventions and school contexts.

**Originality/value:**

This paper adds to the limited evidence base on school-based cooking interventions by theorising how cooking interventions relate to school settings, and how they may achieve integration.

## Introduction

Promoting healthy eating among children is a public health priority amid concern regarding the world wide prevalence of obesity and poor diets (World Health Organisation, 2003). Promoting healthy diets and food choices at an early age is important because childhood eating habits are often sustained into adulthood (Brown and Ogden, 2004), and poor diet is associated with a range of health problems, including diabetes, coronary heart disease and cancers (Kopelman, 2007).

Schools are an important setting for interventions to improve children’s dietary choices (James *et al*., 2007; Sharma, 2006) and the nutritional quality of the food they eat (Waters *et al*., 2011; Young, 1993). School-based health promotion activities can have high reach, and can influence health behaviours such as food consumption through curriculum-based activities and the school environment (Bonell *et al*., 2013; Jamal *et al*., 2013), including the food pupils eat at school (Haynos and O’Donohue, 2012).

The teaching of cooking in schools has been identified as an important aspect of promoting healthy eating, with food and practical cooking skills now compulsory for 5-14-year-olds in parts of the UK (Department for Education, 2013a, b; Welsh Assembly Government, 2009). Cooking sessions aim to give individuals the ability and confidence to prepare, and the inclination to eat, homemade, healthy food (Fisher *et al*., 2011; Hyland *et al*., 2006; Knai *et al*., 2006; Nixon *et al*., 2012; Perez-Rodrigo and Aranceta, 2001, 2003). Preparation and tasting activities can introduce participants to healthy foods (Jones *et al*., 2012; Walters and Stacey, 2009), which is important because consumption of new food types may be linked to repeated exposure (Knai *et al*., 2006; Liquori *et al*., 1998). Interactive cooking sessions also create opportunities for adults to role model and reinforce target behaviours and techniques (Nixon *et al*., 2012). Involvement of parents/families in school-based cooking interventions has been advocated, since this can increase the intensity of intervention processes and exposure to new food types (Knai *et al*., 2006). School cooking activities have the potential to impact on families’ eating practices, through children developing the confidence to request to cook or eat new foods at home (Caraher *et al*., 2013), and by sharing the food they have produced with family members (Drummond, 2010; Hyland *et al*., 2006), though further research is needed to understand the complexities of how children negotiate and control food in the family context (O’Connell and Brannen, 2004).

### Interventions as sociotechnical networks

The development and evaluation of school-based cooking activities and interventions therefore have the potential to make an important contribution to current efforts to improve children’s dietary behaviours and nutritional outcomes. Bisset et al. (2009, 2013) and Hawe et al. (2009) argue that interactions between interventions and settings are integral to the process through which interventions take form, and that settings such as schools should be conceptualised as complex systems. Behaviour change interventions in settings such as schools therefore need to move beyond the linear dissemination of individual-level health messages, to engage with “the dynamics of the context or system” (Hawe *et al*., 2009, p. 269).

Drawing on actor network theory, Bisset et al. (2009) have conceptualised interventions as sociotechnical networks to understand how schools might make use of externally delivered health promotion programmes, and the extent to which they are sustained over the long term. They argue “that programs take form during their implementation through the process of building […] connections” with local actors – such as pupils, school staff and parents, hence the use of the “network” as an organising framework (pp. 556-557). To forge these connections and networks, interventions need to align their goals with those of schools, whereby intervention “goals take form interactively with the interests of programme participants […] and stakeholders” (p. 554). Two key processes help achieve engagement of local actors within a sociotechnical network. First, interventionists must define a problem (problematisation), its relevance to school-based actors, and how their intervention has a role in addressing it. The goals and interests of teachers, pupils and other local actors may therefore need to be integrated within the aims of the intervention. Second, interessment refers to interventionists’ descriptions of the “concrete” ways in which they can address the problem, and the generation of interest in, and commitment to, an intervention on the part of pupils, teachers, etc. Sociotechnical networks comprise both social (human) interactions, and non-human (technical) components – e.g. timetables, instructions, equipment (and in the current study a vehicle containing a mobile classroom), which interventions use as “devices of interessment”.

Key to understanding whether interventions bring about change is the extent to which they expand their sociotechnical network (achieved through the “expansion” and “stabilisation” of connections), which happens when organisations (here school contexts) integrate and routinise aspects of the intervention (including non-human or technical entities such as cooking equipment) within their everyday work, therefore acting on behalf of the intervention (Bisset *et al*., 2009, 2013; Hawe *et al*., 2009).

### School-based cooking interventions

Despite the importance of cooking activities in school to broader healthy eating initiatives, Caraher et al. (2013) suggest that the literature is “sparse and mostly descriptive and not evaluative” (p. 51), whilst a systematic review of cooking interventions in schools identified only four studies that met its quality inclusion criteria (Caraher *et al*., 2010). Studies have found evidence of impacts of cooking interventions on knowledge (in relation to healthy diet and cooking), increases in pupils’ confidence to cook, and higher levels of consumption of, or preference for, healthy foods such as vegetables (Caraher *et al*., 2010, 2013; Liquori *et al*., 1998; Perez-Rodrigo and Aranceta, 1997; Townsend *et al*., 2006). Caraher *et al.* (2010) highlight limitations in those studies which have measured outcomes from school-based cooking interventions, including the tendency to examine perceived ability rather than specific skills, the absence of studies measuring long-term as opposed to short-term effects, and the fact that cooking activities often form part of broader interventions, with the cooking components being poorly described and evaluated.

Few studies have evaluated the implementation of cooking interventions in schools, including the factors which affect schools’ adoption of interventions (Diker *et al*., 2011) or the perspectives of pupils (Lukas and Cunningham-Sabo, 2011; Caraher *et al*., 2010), teachers or parents/carers on intervention content or outcomes. These are important research gaps given the potential for cooking interventions to promote healthy diets, the significant investment in cooking-based projects (Caraher and Seeley, 2010), and the complex social processes through which cooking may generate behaviour change. However previous studies have identified school-level barriers to delivering cooking lessons. These include lack of time (Drummond, 2010), including the marginalisation of cooking within school curricula (Stitt, 1996), teachers’ confidence or comfort with teaching cooking (Diker *et al*., 2011), training (Caraher and Seeley, 2010), and lack of appropriate facilities/materials (Caraher and Seeley, 2010; Diker *et al*., 2011).

One approach to addressing these school-level barriers is the provision of mobile cooking classrooms, which give schools access to specialist teachers and facilities. In the UK a number of Cooking Buses are used to perform this function, with the aim of promoting food hygiene, basic cooking skills and healthy eating, and providing practical skills sessions for pupils, parents and teaching staff. Evaluations of the Cooking Buses in Scotland and England have found qualitative evidence of perceived increases in individual participants’ knowledge, skills and cooking confidence, and use of healthy recipes taught (COI Communications on behalf of the Food Standards Agency, 2004; Margaret Reid Research and Planning, 2012). The Scottish findings also suggested some longer term impacts at the school level – such as the setting up of cooking clubs, but that “legacy plans varied greatly and were inconsistently implemented” (p. V). However, the evidence base for these interventions remains weak in relation to long-term outcomes, intervention change mechanisms, and factors affecting their implementation.

Whilst the provision of mobile cooking classrooms is designed to address school-level barriers to cooking, the use of external agencies to deliver health interventions in schools raises important questions about how they are integrated with existing activities (Segrott *et al*., 2013; Smith *et al*., 1992). Buckley and White’s (2007) review of external agencies’ role in school-based substance misuse education found that “there is little evidence about how schools make use of such [external] contributors, what they do in the classroom, the quality of their contribution and the impact they have on pupils’ knowledge, skills and behaviour” (p. 43). They identify five key implementation issues: the need to integrate external contributions with the curriculum; provision of effective support from schools for the intervention provider; the quality of teaching; whether schools undertake follow-up work; and the resources which external agencies give schools access to.

In this paper we report findings from an evaluation of the Cooking Bus in Wales – a peripatetic cooking classroom capable of hosting up to 16 participants which is used to deliver cookery lessons in schools. The overall aim of the research is to further understanding of how external cooking interventions are implemented in school settings, and this paper focusses on three issues. First, we discuss the aims which schools had for their Cooking Bus visit, and the degree of alignment between these and the Bus’ stated aims. Second, we examine the extent to which the intervention strengthened connections between staff, pupils and parents with cooking. Third, we consider how schools integrated cooking activities into their everyday work following the Bus visit. We use the sociotechnical network framework to theorise these processes and to understand the implementation of the Cooking Bus intervention within complex school systems.

## Method

### The Cooking Bus in Wales intervention

In Wales, UK a Cooking Bus was launched in 2006, commissioned and funded by the Welsh Government as part of its Food and Fitness Implementation Plan (Welsh Assembly Government, 2006), and operated by Design Dimension Educational Trust as part of the Focus on Food campaign (www.focusonfood.org/home.html). The Bus comprised an articulated lorry containing a cooking classroom, fully equipped with cookers, sinks and utensils, etc. (further details are available at: www.focusonfood.org/cooking_buses).

Its aims (see the below list) focussed mainly on the cultivation of cooking skills and knowledge relating to healthy diets (pupils and parents/carers), training and motivating teachers to deliver cooking in the classroom, and the provision of a set of cooking equipment (COOKIT) for use in teaching cooking after the visit. The COOKIT comprised most of the equipment which a teacher would need to teach “practical cooking” sessions for groups of pupils, including jugs, bowls, knives, scales, chopping boards, baking trays, pans, utensils and resources for teachers (www.focusonfood.org/equipment).

Aims of the Cooking Bus in Wales:

- Support the Health Challenge Wales initiative.
- Target Communities First areas to improve diet and help the Government meet dietary targets.
- Support something happening in the school.
- Teach cooking skills, healthy eating, food safety and hygiene.
- Make cooking fun.
- Raise awareness of the importance of a good diet and what they (children) need to eat (including fresh ingredients) and why they need to eat it.
- Show parents how to get food as cheaply as possible and help them source local produce.
- Highlight the importance of fruit and vegetables in their (children’s) diets.
- Motivate teachers and teaching assistants, and children and parents.
- Train teachers to deliver cooking skills and feel confident, and competent.
- Raise the profile of food teaching in schools (and the community) in Wales.
- Provide a practical cooking experience for pupils.
- Make children aware of where food comes from.
- Give the COOKIT to schools, with the expectation that this would help them to take forward cooking activities after the Bus visit.

The Cooking Bus intervention delivered sessions in approximately 40 primary schools annually. These visits targeted schools in the Welsh Network of Healthy School Schemes (Public Health Wales, 2017) and which were located in Communities First areas (Welsh Government, 2013). In addition to pupils’ sessions and inservice training (INSET) sessions for teaching staff, the Bus was used to offer sessions for members of the community, normally parents/carers of pupils in the schools visited.

Three “threads” ran through each session – food hygiene, cooking skills and healthy eating. Pupils’ and community sessions covered similar material, tailored to the age/ability of the participants. The INSET session focussed on training teachers and demonstrating the COOKIT. Cooking Bus sessions comprised cooking demonstrations, followed by opportunities for pupils to prepare age-appropriate dishes, and practise skills (such as cutting with knives). Sessions, lasting approximately one hour, included opportunities to taste different foods. Information giving and discussion on healthy eating, food hygiene, and the technical, social and linguistic aspects of food also formed part of the sessions. Community and staff INSET sessions followed a similar format.

Cooking Buses with similar aims operate in England and Scotland, but each has slightly different remits. The Bus in Scotland (currently funded by the Food Standards Agency and Scottish Government) visits primary and secondary schools and community venues, whereas the main focus of the Bus in Wales was primary schools.

### Research design

Multiple methods, comprising a postal questionnaire to all schools which had received a Bus visit and case studies of the Bus’ work in schools, were used to obtain information from school staff.

### Postal questionnaire to schools

The questionnaire was intended to obtain information about each stage of the visit, including schools’ aims for their visit, the period when the Bus was on the school site, and the period after the Bus left, including the perceived impact of its visit, and whether the COOKIT had been utilised. The questionnaire was piloted with a school which had received a Cooking Bus visit. The final version was sent to staff in each school (including the person that had coordinated the visit) that had received a visit from the Bus prior to 17 May 2008. It comprised a total of 47 items, which were a mix of open-ended, Likert scale and “multiple choice” questions. A summary of relevant “closed” questions is provided in Table I.

### Case studies

Case study methodology – which provides a framework for the study of complex phenomena within their “real life context”, was used to understand how the intervention interacted with school contexts (Yin, 1999). Multiple methods were used in order to describe as fully as possible the influences on the implementation of the Cooking Bus (Inchley *et al*., 2000).

Five school case studies were conducted. In each school four Cooking Bus sessions were observed (the INSET, community and at least two pupil sessions) using a structured guide (Spradley, 1980), to examine the implementation of the intervention activities. Issues considered during observation were: general arrangements and details of staff and pupils present; session content; information given on, and achievement of aims and objectives; participant response; and the interaction between pupils, and between pupils and Cooking Bus staff. In each school up to five semi-structured interviews with teaching and other school staff were conducted (see Table II).

Interviews examined: schools’ aims for their Cooking Bus visit and how schools intended the bus to link with existing activities in school, organisation of the visit, perceived outcomes from the Cooking Bus visit for staff and pupils, and schools’ plans to develop cooking activities as a result of their Bus visit. Discussion groups were also held with pupils and parents/carers, but are not discussed in this paper.

School selection started with the first school in South Wales to receive a visit during the data collection period (due to the study’s limited time period). Schools were then selected to represent a range of schools by size, geographical location (rural and urban) and language status (English/Welsh medium) which received a Cooking Bus visit.

### Data analysis

Interviews were recorded and transcribed. Qualitative data underwent thematic content analysis (Bowling, 1994), with observation, interview and discussion group guides used to create an analytical framework. Different sources of data from each case study (observation data, staff interviews and discussion groups with pupils and parents/carers) were triangulated to understand variation in implementation and schools’ engagement (Miles and Huberman, 1994). Quantitative questionnaire responses were entered into SPSS and descriptive statistics were produced to summarise key characteristics/patterns in the data (Gaddis and Gaddis, 1990). Questionnaire findings were integrated with qualitative data from the case studies to achieve a full understanding of schools’ goals for their visit, the alignment of intervention aims and schools’ goals, and the extent to which cooking activities were sustained and routinized in schools after Cooking Bus visits.

### Confidentiality and ethics

Ethical approval was given by the Cardiff School of Social Sciences Ethics Committee, Cardiff University. Electronic records linking school staff names to ID numbers were held in a separate database to the quantitative data. ID numbers were used instead of names during qualitative data collection.

In case study schools, information sheets were provided to all parents of pupils who were due to visit the Cooking Bus, giving them the option of withdrawing their child from data collection (either observation of Cooking Bus sessions and/or participation in discussion groups). Pupils were provided with age-appropriate information and given the opportunity to refuse consent to be observed during Cooking Bus sessions. Written consent/assent was obtained from school staff, parents and pupils for their participation in interviews/discussion groups.

## Findings

### Study schools

Of the 76 schools that had already received a Cooking Bus visit and which were asked to complete the study questionnaire, 71 agreed. Reasons for non-participation were documented, and included lack of time due to other commitments, and the length of time since the Bus visited. In total, 51 of the 71 schools which agreed to participate in the study returned at least one completed questionnaire.

The five case study schools ranged in size from 93 to 427 pupils, represented urban and rural settings, and included one Welsh medium school (a school in which all education is delivered through the Welsh language). Case study school 1 was situated in an urban area of the South Wales valleys, with a pupil population of 327, and a Free School Meal (FSM) entitlement rate of 51 per cent. School 2 was located on the edge of a small town in rural West Wales (203 pupils, and a FSM rate of 31 per cent). School 3 was in a small north Wales town (479 pupils, FSM rate 21 per cent). The fourth school was a Welsh medium school in rural mid Wales (240 pupils, FSM rate 13 per cent), and shared its visit with a second school. The final school was located on the Isle of Anglesey in North West Wales, and had 93 pupils, and a FSM rate of 23 per cent.

### Aligning intervention aims with schools’ goals

Overall there appeared to be a close alignment between what schools wished to achieve from their Cooking Bus visit and the aims of the Bus intervention. The majority of schools in the study wanted a visit because of the potential impacts on pupils’ dietary behaviours and cooking skills, both of which were central to the Cooking Bus pupil sessions (see the list – Aims of the Cooking Bus in Wales). Over half of questionnaire respondents, and staff in all case study schools, identified that they wanted the Cooking Bus visit to promote healthy eating to pupils as part of the Healthy Schools Scheme. In interviews, staff in all schools felt it would complement nutritional approaches already taking place in school such as fruit tuck shops, improved school lunches, the Welsh Government Free Breakfast Initiative and access to drinking water. The other major reason given was the hope that pupils would learn cooking skills and how to prepare healthy meals. This was identified by 39 of the 68 questionnaire respondents (in 15 of 35 schools) who indicated that the aims of the Bus visit to their school had been agreed. It was echoed by staff in all of the case study schools. Case study schools 2 and 5 wanted to strengthen links between food grown in the school garden with the production of healthy meals, thereby complementing the sustainability message and linking with their Eco Schools project. In Case study schools 2 and 4, teachers felt that the visit of the Cooking Bus would provide the opportunity to develop generic skills identified within the skills framework, such as communication and working together. A reason given by two questionnaire respondents was the hope that the Bus’ visit would provide a forum to demonstrate multi-cultural foods and address cultural, gender and equality issues. Head teachers in Case study schools 1 and 5 wanted the Bus visit to provide a high profile “event” that was enjoyable and memorable for pupils. In total, 14 questionnaire respondents also wanted children to be involved in a fun activity and to give them enthusiasm for cooking.

Many schools hoped that school staff would benefit from the visit through forging new connections with cooking, and this was closely aligned with the Cooking Bus intervention’s stated aims of motivating and training teachers. The importance of developing staff skills to teach cooking within the curriculum and in cooking clubs was identified in the case studies and by questionnaire respondents. In total, 26 questionnaire respondents (total *n* = 96) hoped the visit would equip teachers with enthusiasm, confidence and skills, and a number of staff in case study schools identified the revised design and technology curriculum and the new Foundation Phase national curriculum for three-to-seven-year-olds in Wales (both of which include cooking components) as creating a need for development in this area. In Case study schools 2 and 4, teachers hoped the visit would prepare them to write their food and fitness policies (an action required by the Healthy Schools Scheme).

Involvement of the wider community was mentioned only in a minority of cases. Staff in Case study schools 1 and 5 and 15 questionnaire respondents (total *n* = 96) hoped that the Cooking Bus visit would promote healthy eating messages to families and the local community. Staff in Case study school 3 had the aim of improving the packed lunches that pupils brought to school. Questionnaire respondents representing four schools (total *n* = 35) felt that the purpose of the visit was to encourage parental involvement in cooking with children. The coordinator in Case study school 1 thought that the Bus visit would raise the profile of the school in the local community and provide an opportunity for positive press coverage. Documentary analysis identified that schools were not provided with detailed information about the community session, and fewer questionnaires respondents (34 per cent) had clear expectations about the aim of the session compared with pupils’ (58 per cent) and INSET sessions (57 per cent) (Table I).

The Bus staff, therefore, appeared to achieve alignment of intervention goals with those of local school actors. The Bus’ visit was perceived by school staff as offering practical ways of strengthening or complementing existing school food-related activities, or creating new opportunities for skill development. Pre-visit documentation – a technical component of the network, circulated between the Bus staff and schools, but there were no materials designed to reach parents or draw them into the network. The majority of questionnaire respondents and case study participants did not view the Cooking Bus visit primarily as a means to engage parents, focussing more on pupils and staff.

### Strengthening the connections between schools and cooking

Overall, school staff believed that the aims which they had set for their Bus visit had been achieved. They suggested that the visits raised the profile of food, cooking and healthy eating within schools, and generated interest and enthusiasm among pupils. In Case study school 1, the in-school coordinator emphasised the value of external support, describing how the Bus visit had given “a huge boost to what’s in place. We couldn’t have introduced this aspect [cooking] without outside help”.

New or reinforced connections between pupils, staff and cooking were an important way in which schools assessed the value of their Cooking Bus visit. In a number of cases, pupils exceeded staff expectations of their ability to cook/prepare food, and to behave during sessions. The 93 questionnaire respondents felt that pupils had learnt a wide range of skills and knowledge within the sessions related to learning to cook and preparing healthy meals within a safe and hygienic setting. Staff questionnaires and case studies identified that children had also gained or developed a wider set of social skills, particularly around listening, sharing and organisational skills. One teacher described how “they did go into all aspects of cooking, not just doing the recipe, hygiene, you know, and as I said, the healthy eating, balanced diet, working together, sharing things, you know a lot of elements came into just that one session” (teacher, Case study 4).

The promotion of these skills within Cooking Bus sessions was seen by staff as reinforcing key messages and the broad ethos which schools were attempting to promote among their pupils.

The Bus visit was seen to link well into Healthy Schools Scheme activities, and make helpful connections to projects such as school food gardens. In total, 91 per cent of questionnaire respondents felt that pupil sessions supported what was taught in the school curriculum, including work on healthy eating, personal and social education and design and technology. Case study observation revealed cross-curricular references to science (such as the role of yeast in the rising of bread), geography (e.g. where foods were from) and Welsh (use of incidental Welsh during sessions run through the medium of English).

Another theme in the case studies and questionnaires was that the experience of cooking had produced a feeling of achievement amongst pupils, and that it had been motivational and confidence building. Pupils’ own interests and goals were therefore integrated within the “operation” of the intervention (Bisset *et al*., 2009). Some pupils had told their teachers that they might try using Bus recipes at home, and staff felt that the opportunity for pupils to take their own food away was important. Pupils could help shape these new relationships with food and cooking by potentially extending them into the home setting.

Observation of sessions and analysis of questionnaire responses indicated that the Bus offered access to pupils with additional and special educational needs. These children were often able to succeed at performing cooking tasks. They found the emphasis upon practical tasks (rather than activities such as writing/reading) helpful and the teaching methods/style easy to understand. In Case study school 1, a learning support assistant and teaching assistant commented that it was good for the self-esteem of children with special needs as they were able to answer questions on healthy eating and nutrition on the Bus because these topics had been covered in the curriculum.

One limitation identified by school staff in both questionnaire responses and the case studies was the number of pupils that the Cooking Bus staff could work with during a visit. This was particularly an issue when the Bus visited a large school and there was insufficient time for all pupils to participate in a session. However, despite some misgivings, in answer to the question “Did enough pupils visit the Cooking Bus to achieve the aim(s) of the visit?”, 87 per cent of questionnaire respondents answered “yes”. The concerns expressed by a minority related to the desire to offer a more equitable experience and to prevent upset to pupils. The coordinator in Case study school 1 (a large school) felt that if all pupils had visited the Bus “[…] it would have been a much larger platform for discussion work and follow-up […]”. Another teacher in the same school also “found it strange that they were there for the week, but they didn’t have any sessions on the Monday or Friday. […] you would have got even more children through”.

School staff in case study schools reported feeling more confident and enthusiastic about teaching cooking in the classroom as a result of their engagement with the Bus visit. Provision of an INSET session appeared to play an important part in these processes. One teacher described how “I feel more confident in delivering that part of the curriculum now […]. Even that short INSET was beneficial”. This was endorsed by the majority of questionnaire respondents who suggested that the Cooking Bus visit inspired them to include cooking in planning the curriculum because of their newfound confidence and augmentation of their own expertise, and their observations of the children’s enjoyment of the visit.

Questionnaire responses suggested that attending the INSET session and observing pupils’ sessions had achieved a positive effect on staff development. Staff said they gained practical ideas about how to teach cooking, effective ways of organising lessons, how to deliver basic hygiene lessons, the sorts of recipes/foods that could be prepared and techniques for safely teaching about using equipment. Specific skills included how to teach cutting techniques, ensuring that teaching was age-appropriate and how to link different areas of the curriculum into cookery lessons. In these ways the Cooking Bus staff and sessions therefore appeared to provide practical solutions to issues which teachers were trying to address.

Compared with the impact on pupils, staff and schools as a whole, there was less discussion about what the Bus visits had achieved in relation to the wider community (both in the questionnaire and case studies), suggesting that not all potential actors in the network were engaged. Attendance levels at the community session were often poor (especially parents), ranging from 2 to 15. Case study school 4 did not host a community session due to lack of interest from parents. Schools were provided with limited information about the session to promote it. This was apparent in discussion groups with those who attended the community session. The majority thought they would be taught to cook by watching a demonstration. In Case study school 1, some parents thought that they would be helping to supervise pupils on the Bus. In Case study 5, parents had signed to say they would attend a session but many thought they were giving permission for their children to attend sessions; this meant that very few participants attended. However, other reasons for the poor attendance were reported in interviews and in the questionnaire, including low levels of parental engagement in school activities more generally, and practical barriers to attendance such as work commitments, and childcare requirements. School staff felt that sessions had been effective in raising parental awareness of healthy eating issues, and helping parents develop cooking skills.

One other important theme concerned the pre-visit interactions between the Cooking Bus staff and schools. Much of the interaction between Bus staff and schools prior to visits focussed on the feasibility of housing the Bus on school grounds, and the support which schools would need to provide during the visit in relation to help with washing-up, and information on which groups would be attending (including pupils with special needs) so that sessions could be targeted accordingly. Perceptions of the amount of work needed to prepare for the visit varied widely among questionnaire respondents, often related to whether the Bus was able to fit easily on the school site, and whether the Cooking Bus driver could visit the school to undertake a pre-visit check. Schools appeared to respond to requests for information in different ways, which influenced the amount and complexity of preparatory work they undertook. They also differed in the extent to which they provided information to the Cooking Bus staff about their aims for their visit, or organised help during its time on site, such as arranging for staff/parents to help with washing-up.

For some schools organising the Bus visit was easily achieved (three case study schools and 51 per cent of questionnaire respondents) but problems and challenges were reported by a quarter of questionnaire respondents. Of those reporting problems, 75 per cent said that the needs of the vehicle itself (finding a suitable site with access to water/toilets) represented the greatest challenge. Consequently, schools had different views on the amount of effort required to set up a Bus visit. In total, 41.8 per cent questionnaire respondents reported that arranging the visit took little effort, 41.8 per cent average effort and 16.3 per cent excessive effort. The time required to set up the Bus visit ranged from 0 to 90 hours with a mean of 8.4 hours per school.

### Schools’ future plans for developing cooking activities

Whilst both questionnaire respondents and school staff in case study schools saw the Bus visit as reinforcing existing activities they also felt it was a stimulus for further cooking/healthy eating work in school, suggesting an element of routinizing the intervention within future practice. The new knowledge and positive attitudes towards cooking which pupils developed during their visit to the Bus were seen as important facilitators of future work. Staff believed this would increase pupils’ receptiveness to the introduction of new cooking lessons. They also felt that pupils who had visited the Bus had gained knowledge which future classroom work could build upon, and that these pupils were also likely to instil enthusiasm and interest in the subject amongst other pupils.

Schools had plans to develop cooking activities based on their Bus visit in two broad ways. First, they intended to introduce or increase the teaching of cooking in the curriculum, for example within the design technology curriculum for children aged 7-11 (Key Stage 2). The majority of questionnaire respondents (*n* = 88) reported that their school had introduced cooking classes into the curriculum at least once a term and in some cases weekly. Second, schools planned to strengthen non-curricular cooking activities. The setting up of cooking clubs (or the re-launch of existing ones) was a key example of this, reported by eight questionnaire respondents. In Case study school 1 and in a number of schools which responded to the questionnaire survey, the Bus visit had prompted the staff to think about providing cooking facilities in their school buildings. They had plans to discuss the visit during school assemblies, and for pupils to put together a presentation on healthy food, displays in corridors and inclusion of an article in the newsletter sent home to parents. Other activities included the introduction of “cooking week” at school, and community food lessons. Some teachers had undertaken certification in basic food hygiene in order to be able to teach cooking classes.

Use of the COOKIT – a key aspect of the Cooking Bus network, was central to many schools’ plans and cooking activities, as explained by the head teacher in Case study school 4:

Being a small school, funding is very tight, so having this equipment is going to be tremendous. I would envisage cooking at least once a fortnight and using the skills and the techniques which [Cooking Bus staff] showed the children. You know, we feel as if we’re more confident to get into it more ourselves.

In total, 45 schools which completed the questionnaire indicated they had received a COOKIT, and 36 had used it. The COOKITs made a significant contribution to the schools’ abilities to teach cooking. The INSET session (which demonstrated use of the COOKIT) and the kit itself increased the perceived ability of teachers to work with whole classes or large groups. This was linked to the confidence and ideas they had gained from the INSET session, and the fact that the COOKIT provided suitable equipment for demonstrations and practical skill activities in the classroom. Respondents reported that COOKITs were shared between classes and after school clubs and had been used to support a number of activities, including the preparation of seasonal foods, food for fetes, the local farmers’ market and themed classes. One school had conducted an outreach activity where mothers were invited to cook with Year 2 children to develop basic skills. Where schools intended to develop cooking activities outside of normal classroom activities, the use of the COOKIT was often linked to the creation of new cooking facilities (e.g. physical spaces with cookers, etc.). Two questionnaire participants (from two schools) reported that they had not used their COOKIT because of a lack of suitable facilities.

The main long-term benefits which school staff predicted in relation to the parents’ session were increased involvement with the school, improved parental awareness of healthy eating and that meals could be prepared from scratch (and might be a cheaper option than ready-made meals), better skills for parents and increased support from parents for what children were doing at school, including supporting healthy eating.

## Discussion

This paper has examined key implementation issues in relation to a mobile cooking bus intervention delivered through school visits. A key finding was that the interaction between the intervention and school contexts was important in shaping implementation processes and the potential for the intervention to be sustained by schools over the long-term. Drawing on the concept of the sociotechnical network we have focussed on the degree of alignment between intervention and school goals, the formation of connections between school actors and cooking, and the extent to which school actors had, or planned to, adopt and integrate cooking activities after the Bus had left.

Cooking Bus staff succeeded in aligning the intervention’s goals with those which schools had for the development of cooking activities. Schools’ concern with promoting cooking skills and positive attitudes towards healthy food among pupils were a central aim of the Cooking Bus sessions. Schools also identified a desire to build upon or develop existing healthy eating-related activities (such as school gardens), and develop skills and confidence among teachers to deliver cooking in the curriculum, and through other activities such as clubs. The Cooking Bus intervention offered a way of achieving these goals, and schools appeared to be open to external support to address them. The INSET session – which focussed on teachers’ skills and confidence and the integration of cooking into the curriculum and other aspects of school life, was therefore a key mechanism through which the intervention addressed school-level barriers to undertaking cooking in schools, built connections with teachers, and aligned itself with their interests and concerns.

Cooking Bus sessions promoted aspects of “hands on” cooking which have been identified as important in shaping young people’s dietary behaviours, including cooking skills, self-confidence in using these skills and exposure to healthy foods (Fisher *et al*., 2011; Hyland *et al*., 2006; Jones *et al*., 2012; Knai *et al*., 2006; Nixon *et al*., 2012; Perez-Rodrigo and Aranceta, 2001, 2003; Walters and Stacey, 2009). Through these sessions the Bus intervention might be seen as promoting cooking through creating new connections between pupils and food, and harnessing young people’s goals (e.g. the desire to impress family members with the food they had made) to achieve the intervention’s own aims (promoting healthier diets). Harnessing the goals of local participants in this way is important because it can potentially build commitment and support for the intervention (Bisset *et al*., 2009).

Whilst the sociotechnical network developed relatively strong and stable connections with school staff and pupils, its connections beyond the school gates were less extensive. The relatively low levels of attendance at the community session by parents suggests that potential attendees did not view it as addressing a practical issue or problem for which they were seeking solutions – though it was also perhaps because schools were not provided with detailed information or promotional leaflets for the community session. Practical barriers to attendance (e.g. childcare commitments) may also have played a part. These findings mirror those of Bisset and Potvin (2007), whose study of the Little Cooks-Parental Networks programme found that “While the nutrition education techno-gram was well developed with a clear and understandable objective among the nutritionist and educational networks, the techno-grams which would allow the program to create and/or link to parent and community networks were not clearly defined nor agreed upon” (p. 743). To a large extent parents remained outside the sociotechnical network of the Cooking Bus. However, low levels of parental engagement may also have reflected the more general challenges of engaging parents in school-based activities (Burchett, 2003; Sormunen *et al*., 2012).

Many of the actual and planned processes set off by the Bus visit appeared to function at the system level. How schools intended to build upon and sustain the Cooking Bus visit could potentially be as important as how many individual children received a session during its visit, which was a short, “one off” event rather than a series of longer term interactions. For instance teachers described how they had integrated cooking within the curriculum, and had established cooking clubs. In these ways, the intervention’s sociotechnical network had succeeded in expanding into school systems, through making and stabilising connections with school staff who “invest[ed] and contribute[d]’ to its form (Bisset *et al*., 2009, p. 566). As Bisset *et al.* (2013) suggest, “health professionals [need] to engage in relationship building activities that center less upon passing health promotion messages and building individual skills, and more upon negotiating with a range of stakeholders to learn how intervention goals can be strategically advanced” (p. 17). The ongoing use of the COOKITS was an important part of the Cooking Bus’ integration in the school setting.

The Cooking Bus intervention, whilst popular with schools, and visually appealing, had limited reach in terms of the number of schools it could serve, how many pupils could receive lessons in each school, and the limited capacity to provide follow-up visits. This was a key factor in the recommendation to discontinue its funding in Wales by a recent review of health improvement initiatives (Public Health Wales, 2013). The use of a large vehicle to deliver the intervention created a considerable amount of work for some schools in relation to assessing the suitability of school sites to host the Bus, ensuring its practical needs were met, and deciding which pupils would visit it[1]. A key question is whether the technical aspects of the intervention’s sociotechnical network (the Bus itself in terms of the kitchen it houses and its visual appeal) are essential components of the intervention, or whether there are cheaper methods with greater reach which could achieve similar aims.

Our findings have implications for the design of cooking interventions in schools, and school health promotion programmes by external agencies more generally. The sociotechnical network framework employed in this paper explains some of the processes which might facilitate the integration of externally delivered interventions within school systems. The framework highlights the value of understanding how interventions take shape through their interaction with school systems (Bisset *et al*., 2009), and of building this into the design of interventions. There is also a need for evaluations of external interventions in schools to study how intervention-context interactions shape intervention processes and outcomes. Doing so can help us move beyond simply assessing “uptake of the intervention’s components within a system” (Hawe *et al*., 2009, p. 43) to explain more fully why implementation processes vary across different school systems.

A key implication from our study is that external agencies need to consider carefully the alignment of their goals with those of school actors (Bisset *et al*., 2009), as this can increase the likelihood that schools adopt and integrate intervention goals. If practices and behaviours promoted by external interventions are to be sustained, schools need to perceive their value and relevance, and be willing to integrate them within their everyday work. Commitment from school staff and the compatibility of an intervention with school routines and ethos are important influences on the extent to which interventions are successfully implemented (Audrey *et al*., 2008). Brief, external interventions which lack relevance to schools’ goals, or which are difficult to integrate and adopt, are unlikely to have meaningful impacts. The Cooking Bus intervention’s goals appeared to have broad relevance and appeal to schools, and the alignment of goals happened by “default”. However, other interventions – perhaps those which have less immediately obvious links to the curriculum or schools’ broader mission/ethos, may need to identify at an early stage how they can address issues/goals which matter to individual schools, and the best way to communicate their ability to do this. Our study also highlights the importance of developing effective strategies to engage parents and the wider community into school-based interventions. Intervention teams need to provide schools with clear information about the aims of activities they provide for parents, and ensure that schools are given appropriate materials which they can share with parents to encourage attendance. Identifying and addressing parents’ own goals and the issues which have personal meaning for them and integrating these into the intervention could provide a useful recruitment tool. For instance, parent involvement in interventions could be framed around involvement in pupils’ learning and school life more generally, rather than the teaching of skills to parents.

Interventions need to build support and commitment from school systems if they are to be sustained over the long term. Schools are complex systems, and interventions aimed at effecting school-level change need to be theorised as “events in systems” rather than focused on individual-level “health messages” (Hawe *et al*., 2009). Interventions which focus narrowly on the provision of individual information to pupils, or “one off” class-based sessions are unlikely to have a sustained impact on health behaviours. Whilst external agencies can offer valuable contributions to the teaching of cooking in schools, it is important to understand how they interact with school systems and to identify the processes through which they may strengthen long-term efforts by schools to promote healthy eating among their pupils.

## Figures and Tables

**Table I:** Summary of responses to questionnaire

	Frequency	Valid %
*Prior to the visit, were the aims of the visit as a whole agreed?*		
Yes	68	64.8
No	7	6.7
I don’t know	30	28.6

*To what extent were these aims achieved (visit)?*		
Partially	7	9.9
Completely	64	90.1

*Prior to the visit, were the aims of the pupil session agreed?*		
Yes	58	92.1
No	5	7.9

*To what extent were these aims achieved (pupils session)?*		
Partially	6	9.8
Completely	55	90.2

*Prior to the visit, were the aims of the inset session agreed?*		
Yes	57	57.0
No	10	10.0

*To what extent were these aims achieved (inset session)?*		
Partially	2	3.2
Completely	60	96.8

*Prior to the visit, were the aims of the community session agreed?*		
Yes	32	34.4
No	8	8.6
I don’t know	53	57.0

*To what extent were these aims achieved (community session)?*		
Partially	4	11.1
Completely	32	88.9

*Did enough pupils visit the Cooking Bus to achieve the aim(s) of the visit?*		
Yes	86	86.9

*Did the pupil sessions support what pupils are taught through the curriculum?*		
Yes	92	91.1
I don’t know	9	8.9

*If you have already received your COOKIT, has your school used it?*		
Yes	66	61.7
No	18	16.8
I don’t know	13	12.1

*School role of recipient*		
Headteacher	4	3.7
CB contact	17	15.9
Head teacher and CB contact	22	20.6
Other member of staff	64	59.8

**Table II:** Interviews conducted in case-study schools

School	School/Bus staff interviews
Case study 1	Healthy school coordinator/Cooking Bus coordinator Key Stage 1 teacher who attended pupil and INSET sessions Teachers’ assistant who ran breakfast provision in school Key Stage 2 teacher attended INSET session Headteacher Teacher assistant who attended the parent/community session and a pupil session
Case study 2	Headteacher (did not attend any sessions) Teachers’ assistant (helped in Year 3/4 sessions) Special needs teacher (Autistic Unit) attended INSET session Key Stage 2 teacher attended INSET session Deputy Head teacher and organiser of visit attended Year 6 session
Case study 3	Headteacher attended a pupil session Key Stage 1 teacher/healthy schools coordinator who attended INSET session Deputy head/Key Stage 2 teacher attended a pupil session and INSET session Key Stage 2 teacher attended a pupil session and INSET session Learning support assistant – a parent and attended pupil sessions The headteacher of the school where the Cooking Bus was sited
Case study 4	Headteacher Deputy head teacher and healthy school coordinator Year 4/5 Teacher Year 1/2 Teacher
Case study 5	Headteacher (organiser of the Cooking Bus visit) Year 5 Teacher attended INSET session, observed Year 5 session and organised a cooking club Reception teacher attended INSET session and also healthy school coordinator Nursery teacher attended INSET session

## Note

1 A number of changes were made to the implementation of the Bus, based on the findings from this research. These included the following: revision of the documentation provided to schools to include the aims of the Bus and each session type, including the community session, and production of invitation letter and poster templates to advertise the latter; greater prominence was given in the documentation to the pre-visit information schools need to provide to the Cooking Bus; development of written guidance for Healthy Schools Scheme coordinators concerning their role in helping select schools for Cooking Bus visits; linking of Cooking Bus visits into actions required of schools as part of the Healthy Schools Scheme; reducing the burden of pre-visit preparation which schools undertake, including greater use of the Cooking Bus driver to conduct visits to assess the suitability of school sites to host the Bus; provision of additional cooking sessions on Mondays/Fridays; development of an information sheet outlining key skills taught to pupils which they can take home to share with their parents/carers; distribution of a follow-up progress evaluation form to schools six to nine months after their visit.
